# The Trigonometric Polynomial Like Bernstein Polynomial

**DOI:** 10.1155/2014/174716

**Published:** 2014-08-27

**Authors:** Xuli Han

**Affiliations:** School of Mathematics and Statistics, Central South University, Changsha 410083, China

## Abstract

A symmetric basis of trigonometric polynomial space is presented. Based on the basis, symmetric trigonometric polynomial approximants like Bernstein polynomials are constructed. Two kinds of nodes are given to show that the trigonometric polynomial sequence is uniformly convergent. The convergence of the derivative of the trigonometric polynomials is shown. Trigonometric quasi-interpolants of reproducing one degree of trigonometric polynomials are constructed. Some interesting properties of the trigonometric polynomials are given.

## 1. Introduction

A century ago Bernstein [1] introduced his famous polynomials by defining

(1) Bn(f;x)=∑i=0n(ni)‍(1−x)n−ixif(in),

where *f* is a function defined on the interval [0,1] and *n* is a positive integer. As Bernstein proved, if *f* is continuous on the interval [0,1] then its sequence of Bernstein polynomials converges uniformly to *f* on [0,1]. Thus Bernstein polynomials are important because a constructive proof of Weierstrass' theorem is given. Later, because the Bernstein polynomials are shape preserving, they were found to have practical applications. Many generalizations of them have been proposed. Very fine brief accounts of the Bernstein polynomials are given in Davis [2] and Phillips [3].

However, there are few results on the constructive proof of trigonometric polynomial sequence approximating continuous function. Some authors are interested in the problem of constructing nonnegative trigonometric polynomials (see [4–6]). Trigonometric interpolation has been considered by Salzer [7] and Henrici [8]. Several other authors have addressed Hermite problems, even for arbitrary points. They were mostly interested in existence questions [9], convergence results, and formulae other than Lagrange's (see [10–13]). Quasi-interpolant on trigonometric splines has been discussed in [14]. In [15], authors approximate continuous functions defined on a compact set *E* ∈ [−*π*, *π*] by trigonometric polynomials. Some problems of geometric modeling are solved better by trigonometric splines. Some types of trigonometric splines have been introduced having different features (see [16–19]). One may use the cosine polynomial sequence {cos⁡*kθ*}  (*k* = 0,1,…, *n*) to approximate a continuous function, but this sequence is not a basis of the trigonometric polynomial space of order *n*.

The purpose of this paper is to construct an explicit sequence of trigonometric polynomials like Bernstein polynomials. Thus, trigonometric polynomials may be used like Bernstein polynomials. It is well known that Bernstein polynomials have many applications and are appropriate for numerical computation. New trigonometric polynomials like Bernstein polynomials provide different expressions for function approximation. We will present a symmetric trigonometric polynomial basis of order *n* and show how it works. Although one can construct trigonometric polynomials via simple ways, via trigonometric kernels, for example, we will construct simpler and more evident trigonometric polynomial which converges uniformly to a continuous function *f* defined on the interval [0, *π*/2]. The problem of reproducing one degree of trigonometric polynomials by trigonometric quasi-interpolants is also solved.

The remainder of this paper is organized as follows. In Section 2, the basis functions of the trigonometric polynomial space are presented and the properties of the basis functions are shown. In Section 3, a sequence of trigonometric polynomials is described and its convergence is discussed. Trigonometric quasi-interpolants of reproducing one degree of trigonometric polynomials are given in Section 4.

## 2. Trigonometric Basis Functions

**Definition 1 .**

For *u* ∈ [0, *π*/2], *n* ∈ *N*, let *s*(*u*) = 1 − sin *u*, *c*(*u*) = 1 − cos⁡ *u*, *w*(*u*) = sin *u* + cos⁡ *u* − 1; one defines trigonometric polynomials of degree *n* as follows:

(2) $$\begin{array}{c} T_{i,n}\left(u\right)=\{\begin{array}{cc} a_{i,n}s^{n-i}\left(u\right)w^{i}\left(u\right), & i=0,1,\ldots ,n,\\ a_{i,n}w^{2n-i}\left(u\right)c^{i-n}\left(u\right), & i=n+1,n+2,\ldots ,2n, \end{array} \end{array}$$

where

(3) $$\begin{array}{c} a_{i,1}=\{\begin{array}{cc} 1, & i=0,1,2,\\ 0, & i\neq 0,1,2, \end{array} \end{array}$$

(4) $$\begin{array}{c} a_{i,n+1}=\{\begin{array}{cc} 0.5a_{i-2,n}+a_{i-1,n}+a_{i,n}, & i\leq n,\\ 0.5a_{i-2,n}+a_{i-1,n}+0.5a_{i,n}, & i=n+1,\\ a_{i-2,n}+a_{i-1,n}+0.5a_{i,n}, & i\geq n+2. \end{array} \end{array}$$

We choose domain [0, *π*/2] in Definition 1 so that *s*(*u*) and *c*(*u*) are monotone, and *w*(*u*) is convex. From (3) and (4), we can obtain the coefficients of the trigonometric polynomials as Table 1.

**Property 2 .**

Linear independence property: the set of the trigonometric polynomials {*T*_0,*n*_(*u*), *T*_1,*n*_(*u*),…, *T*_2*n*,*n*_(*u*)} is linearly independent on [0, *π*/2].

**Proof**

Consider the trigonometric polynomial space

(5) Tn≔span⁡{1,sin(u),cos⁡(u),sin(2u),cos⁡(2u),…,sin⁡(nu),cos⁡⁡(nu)};

we know that

(6) $$\begin{array}{c} \sin^{i}\left(u\right)\cos^{j}\left(u\right)\in T_{n},\;i+j\leq n \end{array}$$

and then

(7) $$\begin{array}{c} T_{k,n}\left(u\right)\in T_{n},\;k\leq 2n. \end{array}$$

On the other hand,

(8) cos⁡(iu)∈span⁡{1,cos⁡(u),cos⁡2(u),…,cos⁡i(u)},sin(iu) ∈span⁡{sin(u),sin(u)cos⁡(u),…,sin(u)cos⁡i−1(u)},sini(u)=[c(u)+w(u)]i,  cos⁡i(u)=[s(u)+w(u)]i,

and 2*s*(*u*)*c*(*u*) = *w* ^2^(*u*); we have

(9) cos⁡(iu),sin(iu)∈span⁡{T0,n(u),T1,n(u),…,T2n,n(u)},i≤n.

Hence,

(10) $$\begin{array}{c} T_{n}=\mathrm{span}\left\{T_{0,n}\left(u\right),T_{1,n}\left(u\right),\ldots ,T_{2n,n}\left(u\right)\right\}. \end{array}$$

Since the set of the trigonometric polynomials {1, sin(*u*), cos⁡(*u*),…, sin(*nu*), cos⁡(*nu*)} is linearly independent, we conclude that the set of the trigonometric polynomials {*T*_0,*n*_(*u*), *T*_1,*n*_(*u*),…, *T*_2*n*,*n*_(*u*)} is linearly independent on [0, *π*/2].

The set of the trigonometric polynomials {*T*_0,*n*_(*u*), *T*_1,*n*_(*u*),…, *T*_2*n*,*n*_(*u*)} forms a basis for the trigonometric polynomial space *T*_*n*_. We refer to the trigonometric functions as trigonometric basis functions.

**Figure 1:**
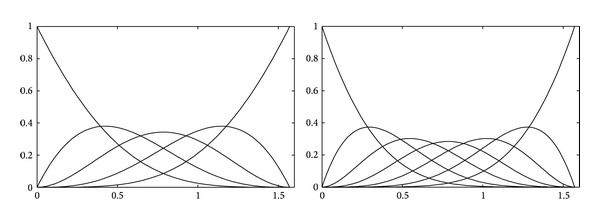
Trigonometric basis functions with *n* = 2 and *n* = 3.

Figure 1 shows the graphs of trigonometric basis functions with *n* = 2 on the left and with *n* = 3 on the right.

Now we show that trigonometric sequence {*T*_0,*n*_(*u*), *T*_1,*n*_(*u*), ⋯, *T*_2*n*,*n*_(*u*)} has different properties than the sequence {1, sin(*u*), cos⁡(*u*),…, sin(*nu*), cos⁡(*nu*)}. Some important properties of the following are useful in the interest of constructing trigonometric polynomial approximants.

**Property 3 .**

Positivity of the basis functions: if *u* ∈ (0, *π*/2), then *T*_*i*,*n*_(*u*) > 0, *i* = 0,1,…, 2*n*.

**Proof**

From (3) and (4), it is easy to see that *a*_*i*,*n*_ > 0 for all possible *i*. Since 0 < *s*(*u*), *w*(*u*), *c*(*u*) < 1, it follows that *T*_*i*,*n*_(*u*) > 0.

**Property 4 .**

Partition of unity for the basis functions: for all *n* ∈ *N*, we have

(11) $$\begin{array}{c} \overset{2n}{\underset{i=0}{\sum }}T_{i,n}\left(u\right)=1. \end{array}$$

**Proof**

Obviously,

(12) T0,1(u)+T1,1(u)+T2,1(u) =s(u)+w(u)+c(u)=1.

We assume that the formula is true for *n*. Since *a*_*i*,*n*_ = 0 for *i* < 0 or *i* > 2*n*, from (2), (4), and *w* ^2^(*u*) = 2*s*(*u*)*c*(*u*) we have

(13) ∑i=02n+2Ti,n+1(u) =∑i=0n(0.5ai−2,n+ai−1,n+ai,n)sn+1−i(u)wi(u)  +(0.5an−1,n+an,n+0.5an+1,n)wn+1(u)  +∑i=n+22n+2(ai−2,n+ai−1,n+0.5ai,n)w2n+2−i(u)ci−n−1(u) =c(u)∑i=0nTi−2,n(u)+w(u)∑i=0nTi−1,n(u)+s(u)∑i=0nTi,n(u)  +c(u)Tn−1,n(u)+w(u)Tn,n(u)+s(u)Tn+1,n(u)  +c(u)∑i=n+22n+2Ti−2,n(u)+w(u)∑i=n+22n+2Ti−1,n(u)  +s(u)∑i=n+22n+2Ti,n(u) =c(u)∑i=02nTi,n(u)+w(u)∑i=02nTi,n(u)+s(u)∑i=02nTi,n(u)=1.

This is (11) with *n* replaced by *n* + 1; the proof is complete.

**Property 5 .**

Symmetry of the basis functions: for *u* ∈ [0, *π*/2], we have

(14) $$\begin{array}{c} T_{i,n}\left(u\right)=T_{2n-i,n}\left(\frac{\pi }{2}-u\right),\;i=0,1,\ldots ,n. \end{array}$$

**Proof**

Obviously, *a*_*i*,1_ = *a*_2−*i*,1_, *i* = 0,1. Assume *a*_*i*,*n*−1_ = *a*_2(*n*−1)−*i*,*n*−1_, *i* = 0,1,…, *n* − 1; from (4) we have

(15) a2n−i,n=a2n−i−2,n−1+a2n−i−1,n−1+0.5a2n−i,n−1=ai,n−1+ai−1,n−1+0.5ai−2,n−1=ai,n,

for *i* = 0,1,…, *n*. These imply that the coefficients of *T*_*i*,*n*_(*u*) are symmetric. Thus, for *i* = 0,1,…, *n*, we have

(16) T2n−i,n(π2−u)=a2n−i,nwi(π2−u)cn−i(π2−u)=ai,nwi(u)sn−i(u)=Ti,n(u).

Based on Property 5, we refer to the basis functions as symmetric trigonometric basis functions.

**Property 6 .**

Recurrence relation of the basis functions: for *n* ≥ 1 and *i* = 0,1,…, 2*n* + 2, we have

(17) $$\begin{array}{c} T_{i,n+1}\left(u\right)=c\left(u\right)T_{i-2,n}\left(u\right)+w\left(u\right)T_{i-1,n}\left(u\right)+s\left(u\right)T_{i,n}\left(u\right), \end{array}$$

where *T*_−2,*n*_(*u*) = *T*_−1,*n*_(*u*) = *T*_2*n*+1,*n*_(*u*) = *T*_2*n*+2,*n*_(*u*) = 0.

**Proof**

From (2) and (4), for *i* = 0,1,…, *n*, we have

(18) Ti,n+1(u)=ai,n+1sn+1−i(u)wi(u)=(0.5ai−2,n+ai−1,n+ai,n)sn+1−i(u)wi(u)=[c(u)ai−2,ns2(u)+w(u)ai−1,ns(u)w(u)  +s(u)ai,nw2(u)]sn−i(u)wi−2(u)=c(u)Ti−2,n(u)+w(u)Ti−1,n(u)+s(u)Ti,n(u),Tn+1,n+1(u)=an+1,n+1wn+1(u)=(0.5an−1,n+an,n+0.5an+1,n)wn+1(u)=[c(u)an−1,ns(u)+w(u)an,nw(u)  +s(u)an+1,nc(u)]wn−1(u)=c(u)Tn−1,n(u)+w(u)Tn,n(u)+s(u)Tn+1,n(u).

For *i* = *n* + 2, *n* + 3,…, 2*n* + 2, we have

(19) Ti,n+1(u)=ai,n+1w2n+2−i(u)ci−n−1(u)=(ai−2,n+ai−1,n+0.5ai,n)w2n+2−i(u)ci−n−1(u)=[c(u)ai−2,nw2(u)+w(u)ai−1,nw(u)c(u)+s(u)ai,nc2()(u)]w2n−i(u)ci−n−2(u)=c(u)Ti−2,n(u)+w(u)Ti−1,n(u)+s(u)Ti,n(u).

**Property 7 .**

Degree elevation: for all *n* ≥ 1, we have

(20) Ti,n(u)=ai,nai,n+1Ti,n+1(u)+ai,nai+1,n+1Ti+1,n+1(u) +ai,n2ai+2,n+1Ti+2,n+1(u)

for *i* = 0,1,…, *n* − 1,

(21) Tn,n(u)=an,nan,n+1Tn,n+1(u)+an,nan+1,n+1Tn+1,n+1(u) +an,nan+2,n+1Tn+2,n+1(u),Ti,n(u)=ai,n2ai,n+1Ti,n+1(u)+ai,nai+1,n+1Ti+1,n+1(u) +ai,nai+2,n+1Ti+2,n+1(u)

for *i* = *n* + 1, *n* + 2,…, 2*n*.

**Proof**

For *i* = 0,1,…, *n* − 1, by (2) we have

(22) Ti,n(u)=ai,nsn−i(u)wi(u)(s(u)+w(u)+c(u))=ai,nsn+1−i(u)wi(u)+ai,nsn−i(u)wi+1(u) +0.5ai,nsn−i−1(u)wi+2(u).

From this we obtain (20). In the same way, we have (21).

**Property 8 .**

Derivative of the basis functions: for *i* = 0,1,…, *n* − 1, we have

(23) Ti,n′(u)=iai,nai−1,nTi−1,n(u)−(n−i)Ti,n(u) −(2n−i)ai,n2ai+1,nTi+1,n(u),

(24) $$\begin{array}{c} T_{n,n}^{\prime }\left(u\right)=\frac{na_{n,n}}{a_{n-1,n}}\left[T_{i-1,n}\left(u\right)-T_{i+1,n}\left(u\right)\right]. \end{array}$$

For *i* = *n* + 1, *n* + 2,…, 2*n*, we have

(25) Ti,n′(u)=iai,n2ai−1,nTi−1,n(u)+(i−n)Ti,n(u) −(2n−i)ai,nai+1,nTi+1,n(u).

**Proof**

For *i* = 0, we have

(26) T0,n′(u)=−nsn−1(u)cos⁡u=−nsn−1(u)(s(u)+w(u))=−nT0,n−T1,n.

This implies the case *i* = 0 of (23). For *i* = 1,2,…, *n* − 1, we have

(27) Ti,n′(u)=ai,n[isn−i+1(u)wi−1(u)−(n−i)sn−i(u)wi(u)−(n−i2)sn−i−1(u)wi+1(u)].

This implies the cases *i* ≠ 0 of (23). In the same way, we can obtain the results on the other cases.

**Property 9 .**

Maximum values: for *i* = 0,1,…, 2*n*, *T*_*i*,*n*_(*u*) obtains its maximum value at

(28) $$\begin{array}{c} u=\arcsin\frac{1}{2n}\left(\sqrt{n^{2}+2ni-i^{2}}+i-n\right). \end{array}$$

**Proof**

Directly derivation computing to (2), we have

(29) Ti,n′(u)=ai,nsn−i−1(u)wi−1(u) ×[is2(u)−(n−i)s(u)w(u)−(n−i2)w2(u)],

for *i* = 0,1,…, *n*, and

(30) Ti,n′(u)=ai,nw2n−i−1(u)ci−n−1(u) ×[0.5iw2(u)−(n−i)w(u)c(u)−(2n−i)c2(u)],

for *i* = *n* + 1, *n* + 2,…, 2*n*. Since *w* ^2^(*u*) = 2*s*(*u*)*c*(*u*), we obtain

(31) Ti,n′(u)=ai,nsn−i(u)wi−1(u) ×[is(u)−(n−i)w(u)−(2n−i)c(u)],

for *i* = 0,1,…, *n*, and

(32) Ti,n′(u)=ai,nw2n−i−1(u)ci−n(u) ×[is(u)−(n−i)w(u)−(2n−i)c(u)],

for *i* = *n* + 1, *n* + 2,…, 2*n*. Let *is*(*u*)−(*n* − *i*)*w*(*u*)−(2*n* − *i*)*c*(*u*) = 0; we have *n*[cos⁡*u* − sin*u*] = *n* − *i* and then

(33) $$\begin{array}{c} \sin u=\frac{1}{2}\left(\sqrt{2-{\left(\frac{n-i}{n}\right)}^{2}}-\frac{n-i}{n}\right). \end{array}$$

From this we obtain (28).

In the proof of Property 5, we have shown that the coefficients of the trigonometric basis functions are symmetric. Now we give further properties of the coefficients of the trigonometric basis functions.

**Property 10 .**

Explicit formula: for the coefficients of the trigonometric basis functions given by (4), we have

(34) $$\begin{array}{c} a_{i,n}=\overset{\left[i/2\right]}{\underset{k=0}{\sum }}\frac{n!}{2^{k}k!\left(n-i+k\right)!\left(i-2k\right)!},\;i=0,1,\ldots ,n. \end{array}$$

**Proof**

Since *a*_0,1_ = *a*_1,1_ = 1, *a*_0,2_ = 1, *a*_1,2_ = *a*_2,2_ = 2, (34) holds for *n* = 1 and *n* = 2 obviously. We assume that the formula (34) is true for *a*_*i*,*n*−1_, *i* = 0,1,…, *n* − 1; then

(35) 12ai−2,n−1=∑k=0[(i−2)/2](n−1)!2k+1k!(n−i+k+1)!(i−2k−2)!=∑k=1[i/2](n−1)!2k(k−1)!(n−i+k)!(i−2k)!,ai−1,n−1=(n−1)!(n−i)!(i−1)! +∑k=1[(i−1)/2](n−1)!2kk!(n−i+k)!(i−2k−1)!,ai,n−1=(n−1)!(n−i−1)!i!+∑k=1[i/2](n−1)!2kk!(n−i+k−1)!(i−2k)!.

By (4), for even numbers 0 ≤ *i* ≤ *n* − 1, we have

(36) ai,n=12ai−2,n−1+ai−1,n−1+ai,n−1=∑k=1[(i−1)/2](n−1)!2kk!(n−i+k)!(i−2k)!    ×[k+(i−2k)+(n−i+k)] +(n−1)!(n−i)!(i−1)!+(n−1)!(n−i−1)!i! +(n−1)!2i/2(i/2−1)!(n−i/2)!+(n−1)!2i/2(i/2)!(n−i/2−1)!=∑k=1[(i−1)/2]n!2kk!(n−i+k)!(i−2k)!+n!(n−i)!i! +n!2i/2(i/2)!(n−i/2)!=∑k=0[i/2]n!2kk!(n−i+k)!(i−2k)!.

For odd numbers 1 ≤ *i* ≤ *n* − 1, we have

(37) ai,n=(n−1)!(n−i)!(i−1)!+(n−1)!(n−i−1)!i! +∑k=1[i/2](n−1)!2kk!(n−i+k)!(i−2k)!    ×[k+(i−2k)+(n−i+k)]=n!(n−i)!i!+∑k=1[i/2]n!2kk!(n−i+k)!(i−2k)!=∑k=0[i/2]n!2kk!(n−i+k)!(i−2k)!.

By induction, the proof is complete.

**Property 11 .**

Recurrence relation of the coefficients: for the coefficients of the trigonometric basis functions given by (4), we have

(38) (i+1)ai+1,n=(n−i)ai,n+12(2n−i+1)ai−1,n,i=1,2,…,n−1

(39) (2n−i+1)ai−1,n=(i−n)ai,n+12(i+1)ai+1,n,i=n+1,n+2,…,2n−1.

**Proof**

For *i* = *n* + 1, *n* + 2,…, 2*n* − 1, by the symmetry of the coefficients shown in the proof of Property 4, we can obtain (39) from (38). Therefore, we consider only the cases *i* = 1,2,…, *n* − 1. By (4), we have *a*_0,*n*_ = 1, *a*_1,*n*_ = *n*. When *i* is an odd number, we have

(40) (n−i)ai,n+12(2n−i+1)ai−1,n =n!(n−i−1)!i!+∑k=1[i/2]n!(n−i)2kk!(n−i+k)!(i−2k)!  +∑k=0[(i−1)/2]−1n!(2n−i+1)2k+1k!(n−i+k+1)!(i−2k−1)!  +n!(2n−i+1)2(i+1)/2((i−1)/2)!((2n−i+1)/2)! =n!(n−i−1)!i!+∑k=1[(i−1)/2]n!(i+1)2kk!(n−i+k−1)!(i−2k+1)!  +n!(i+1)2(i+1)/2((i+1)/2)!(n−(i+1)/2)! =∑k=0[(i+1)/2]n!(i+1)2kk!(n−i+k−1)!(i−2k+1)! =(i+1)ai+1,n.

When *i* is an even number, analogously, we have

(41)   (n−i)ai,n+12(2n−i+1)ai−1,n =n!(n−i−1)!i!+∑k=1[i/2]n!(n−i)2kk!(n−i+k)!(i−2k)!  +∑k=0[(i−1)/2]n!(2n−i+1)2k+1k!(n−i+k+1)!(i−2k−1)! =n!(n−i−1)!i!+∑k=1[i/2]n!(i+1)2kk!(n−i+k−1)!(i−2k+1)! =(i+1)ai+1,n.

By Property 10 or Property 11, we have

(42) a0,n=1,a1,n=n for  n≥1,a2,n=12!n2 for  n≥2,a3,n=13!n(n−1)(n+1) for  n≥3,a4,n=14!n(n−1)(n2+n−3) for  n≥4,  a5,n=15!n(n−1)(n−2)(n2+3n−3)for  n≥5,a6,n=16!n(n−1)(n−2)(n3+3n2−13n)for  n≥6,⋮

and so on.

**Property 12 .**

Positivity of the coefficients: for *i* = 1,2,…, *n* − 1, *n* + 1,…, 2*n* − 1,

(43) $$\begin{array}{c} a_{i,n}^{2}-a_{i-1,n}a_{i+1,n}>0. \end{array}$$

**Proof**

Obviously, (43) holds when *n* = 1,2. For *i* = 0,1,…, *n*, *n* ≥ 3, by (38) we have

(44) ai,n+1=12ai−2,n+ai−1,n +1i[(n−i+1)ai−1,n+12(2n−i+2)ai−2,n]=n+1i(ai−2,n+ai−1,n).

Then, for 4 ≤ *i* ≤ *n*,

(45) 1(n+1)2(ai,n+12−ai−1,n+1ai+1,n+1) =1i2(ai−2,n+ai−1,n)2  −1i2−1(ai−3,n+ai−2,n)(ai−1,n+ai,n) =1i2(ai−2,n2−ai−3,nai−1,n)  +1i2(ai−1,n2−ai−2,nai,n)+i2−2i2(i2−1)ai−2,nai−1,n  −1i2(i2−1)(ai−3,nai−1,n+ai−2,nai,n)  −1i2−1ai−3,nai,n =1i2(ai−2,n2−ai−3,nai−1,n)+1i2(ai−1,n2−ai−2,nai,n)  +i2−2i2(i2−1)ai−2,nai−1,n−1i2(i2−1)ai−2,nai,n  −1i2(i2−1)[i−2n−i+3ai−2,n−2n−i+42(n−i+3)ai−4,n]  ×ai−1,n  −1i2−1[2(i−1)2n−i+3ai−1,n−2(n−i+2)2n−i+3ai−2,n]ai,n =1i2(ai−2,n2−ai−3,nai−1,n)+1i2(ai−1,n2−ai−2,nai,n)  +[(i−2)(n−i+2)i2(i2−1)(n−i+3)+1i(i+1)]ai−2,nai−1,n  +[2(n−i+2)(i2−1)(2n−i+3)−1i2(i2−1)]ai−2,nai,n  +2n−i+42i2(i2−1)(n−i+3)ai−4,nai−1,n  −2(i−1)(i2−1)(2n−i+3)ai−1,nai,n =1i2(ai−2,n2−ai−3,nai−1,n)+1i2(ai−1,n2−ai−2,nai,n)  +[(i−2)(n−i+2)i2(i2−1)(n−i+3)+1i(i+1)(2n−i+3)]  ×ai−2,nai−1,n  +2(i+1)(n−i+1)+2i+1i2(i+1)(2n−i+3)ai−2,nai,n  +2n−i+42i2(i2−1)(n−i+3)ai−4,nai−1,n  −2(n−i+1)i(i+1)(2n−i+3)ai−1,n2 =1i2(ai−2,n2−ai−3,nai−1,n)  +2n+i2+3i2(i+1)(2n−i+3)(ai−1,n2−ai−2,nai,n)  +[(i−2)(n−i+2)i2(i2−1)(n−i+3)+1i(i+1)(2n−i+3)]  ×ai−2,nai−1,n  +2n+3i2(i+1)(2n−i+3)ai−2,nai,n  +2n−i+42i2(i2−1)(n−i+3)ai−4,nai−1,n.

These equalities also hold for *i* = 2,3. When *i* = 1,

(46) $$\begin{array}{c} a_{1,n+1}^{2}-a_{0,n+1}a_{2,n+1}=\frac{1}{2}{\left(n+1\right)}^{2}. \end{array}$$

By induction and symmetry, (43) holds.

## 3. Symmetric Trigonometric Polynomials

### 3.1. The Construction of the Trigonometric Polynomials

We will discuss trigonometric polynomial approximation on the special interval [0, *π*/2] because the change of variable *x* = *a* + 2*t*(*b* − *a*)/*π* can be used to go back and forth between [*a*, *b*] and [0,1].

**Definition 13 .**

Given nodes *x*_*i*,*n*_ ∈ [0, *π*/2], *i* = 0,1,…, 2*n* and function values *f*(*x*_*i*,*n*_) ∈ *R*, we define trigonometric polynomials as follows:

(47) $$\begin{array}{c} T_{n}\left(f,x\right)=\overset{2n}{\underset{i=0}{\sum }}T_{i,n}\left(x\right)f\left(x_{i,n}\right),\;x\in \left[0,\frac{\pi }{2}\right]. \end{array}$$

Since the symmetry of the Trigonometric basis functions, we call (47) as symmetric trigonometric polynomials.

Obviously, *T*_*n*_ is a linear operator. Based on Property 3, another property of these operator is that they are positive. This implies that if *f* ≥ 0, then *T*_*n*_(*f*, *x*) ≥ 0.

For computing conveniently, we can choose nodes *x*_*i*,*n*_ = *i*/(2*n*). On the convergence of *T*_*n*_(*f*, *x*), two kinds of the nodes will be discussed. One kind of the nodes is *x*_0,1_ = 0, *x*_1,1_ = *π*/4, *x*_2,1_ = *π*/2, and

(48) xi,n={arcsin(0.5ai−2,n−1+ai−1,n−1ai,n), i=0,1,…,n−1,π4, i=n,π2−x2n−i,n, i=n+1,n+2,…,2n,

for *n* > 1. Another kind of the nodes is *x*_0,1_ = 0, *x*_1,1_ = *π*/4, *x*_2,1_ = *π*/2, and

(49) xi,n={arcsin(0.5ai−2,n−1+ai−1,n−1Ai,n), i=0,1,…,n−1,π4, i=n,π2−x2n−i,n, i=n+1,n+2,…,2n,

for *n* > 1, where

(50) $$\begin{array}{c} A_{i,n}=\sqrt{{\left(0.5a_{i-2,n-1}+a_{i-1,n-1}\right)}^{2}+{\left(a_{i-1,n-1}+a_{i,n-1}\right)}^{2}}. \end{array}$$

We can also rewrite

(51) $$\begin{array}{c} \frac{1}{2}a_{i-2,n-1}+a_{i-1,n-1}=\frac{2n-i+1}{2n}a_{i-1,n},\\ a_{i-1,n-1}+a_{i,n-1}=\frac{i+1}{n}a_{i+1,n}. \end{array}$$

By Property 11, expression (48) can be changed to

(52) $$\begin{array}{c} \sin x_{i,n}=\frac{0.5a_{i-2,n-1}+a_{i-1,n-1}}{a_{i,n}}=\frac{na_{i-1,n-1}+ia_{i,n-1}}{n\left(a_{i-1,n-1}+2a_{i,n-1}\right)}, \end{array}$$

and (49) can be changed to

(53) $$\begin{array}{c} \tan x_{i,n}=\frac{0.5a_{i-2,n-1}+a_{i-1,n-1}}{a_{i-1,n-1}+a_{i,n-1}} \end{array}$$

for *i* = 0,1,…, *n* − 1. By Property 12, we have

(54) $$\begin{array}{c} \frac{a_{n-1,n}}{a_{n,n}}>\frac{a_{n-2,n}}{a_{n-1,n}}>\cdots >\frac{a_{0,n}}{a_{1,n}}=\frac{1}{n}. \end{array}$$

Therefore, for *i* = 0,1,…, *n* − 1, it is easy to show that the node sequences (48) and (49) are monotonely increasing, respectively. In the following section, we can see that $\sin x_{n-1,n}<\sin x_{n,n}=\sqrt{2}/2$ for (48) or (49).

**Example 14 .**

Let us consider the function as follows:

(55) $$\begin{array}{c} f_{1}\left(x\right)=\cos\left(3x\right)\exp\left(x\right),\;x\in \left[0,\frac{\pi }{2}\right]. \end{array}$$

**Figure 2:**
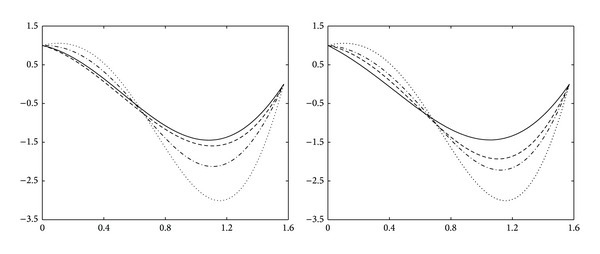
Approximation curves for the function *f*_1_.

Figure 2 shows the approximation curves of this function. On the left of Figure 2, the functional curve (dotted line), the quadratic trigonometric curve (solid line), the quartic Bernstein polynomial curve (dashed line), and the quartic trigonometric curve (dashdot line) are shown with equidistant nodes, respectively. On the right of Figure 2, the functional curve (dotted line), the quadratic trigonometric curve (solid line), the cubic trigonometric curve (dashed line), and the quartic trigonometric curve (dashdot line) are shown with node expression (48), respectively.

**Example 15 .**

Let us consider the function as follows:

(56) $$\begin{array}{c} f_{2}\left(x\right)=\exp\left(-{\left(x-0.2\right)}^{2}\right)+\exp\left(-{\left(x-2\right)}^{2}\right),\;x\in \left[0,\frac{\pi }{2}\right]. \end{array}$$

**Figure 3:**
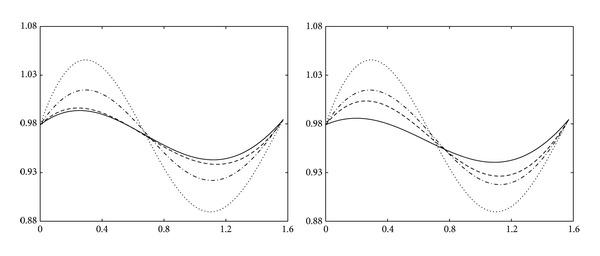
Approximation curves for the function *f*_2_.

Figure 3 shows the approximation curves of this function. On the left of Figure 3, the functional curve (dotted line), the quadratic trigonometric curve (solid line), the quartic Bernstein polynomial curve (dashed line), and the quartic trigonometric curve (dashdot line) are shown with equidistant nodes, respectively. On the right of Figure 3, the functional curve (dotted line), the quadratic trigonometric curve (solid line), the cubic trigonometric curve (dashed line), and the quartic trigonometric curve (dashdot line) are shown with node expression (48), respectively.

### 3.2. The Convergence of the Trigonometric Polynomials

The following theorem will be used repeatedly for the proof of the convergence of the trigonometric polynomials.

**Theorem 16 .**

For the coefficients of trigonometric basis functions, one has

(57) $$\begin{array}{c} \underset{n\to \infty }{\lim}\frac{a_{i-1,n}^{2}-a_{i-2,n}a_{i,n}}{a_{i,n+1}^{2}}=0,\;i=1,2,\ldots ,n, \end{array}$$

(58) $$\begin{array}{c} \underset{n\to \infty }{\lim}\frac{a_{n-1,n}}{a_{n,n}}=\sqrt{2},\;\;\underset{n\to \infty }{\lim}\frac{0.5a_{n-1,n}+a_{n,n}}{a_{n+1,n+1}}=\frac{\sqrt{2}}{2}. \end{array}$$

**Proof**

Obviously, (57) holds for *i* = 1. For *i* = 2,3,…, *n*, by (38),

(59) ai−1,n2−ai−2,nai,n =ai−1,n[n−i+2i−1ai−2,n+2n−i+32(i−1)ai−3,n]  −ai−2,n[n−i+1iai−1,n+2n−i+22iai−2,n] =−2n−i+32(i−1)(ai−2,n2−ai−3,nai−1,n)  +n+1(i−1)iai−2,n(ai−2,n+ai−1,n),

we obtain

(60) $$\begin{array}{c} 0<a_{i-1,n}^{2}-a_{i-2,n}a_{i,n}<\frac{n+1}{\left(i-1\right)i}a_{i-2,n}\left(a_{i-2,n}+a_{i-1,n}\right). \end{array}$$

Then, by (44),

(61) $$\begin{array}{c} \frac{a_{i-1,n}^{2}-a_{i-2,n}a_{i,n}}{a_{i,n+1}^{2}}<\frac{ia_{i-2,n}}{\left(n+1\right)\left(i-1\right)\left(a_{i-2,n}+a_{i-1,n}\right)}. \end{array}$$

Since

(62) (i−1)ai−1,n=(n−i+2)ai−2,n+0.5(2n−i+3)ai−3,n≥(n−i+2)ai−2,n,

we have

(63) $$\begin{array}{c} \frac{a_{i-1,n}^{2}-a_{i-2,n}a_{i,n}}{a_{i,n+1}^{2}}<\frac{i}{{\left(n+1\right)}^{2}}. \end{array}$$

From this we obtain (57).

Let *a*_*n*_ = *a*_*n*−1,*n*_/*a*_*n*,*n*_; we have

(64) an+1=an,n+1an+1,n+1=0.5an−2,n+an−1,n+an,nan−1,n+an,n=(n+1)(an−1,n+2an,n)(n+2)(an−1,n+an,n)=(n+1)(2+an)(n+2)(1+an)

and then, by recursion,

(65) an+1−an =(n+1)(an−1−an)(n+2)(1+an−1)(1+an)+ann(n+2) =n(an−1−an−2)(n+2)(1+an−2)(1+an−1)2(1+an)  −an−1(n−1)(n+2)(1+an−1)(1+an)+ann(n+2) =n(an−1−an−2)(n+2)(1+an−2)(1+an−1)2(1+an)  +an−an−1(n−1)(n+2)(1+an−1)(1+an)  +1n+2[1n−1(n−1)(1+an−1)(1+an)]an.

From (54) we have

(66) $$\begin{array}{c} \frac{1}{n}-\frac{1}{\left(n-1\right)\left(1+a_{n-1}\right)\left(1+a_{n}\right)}>\frac{1}{n}-\frac{1}{n+1}>0,\\ a_{n+1}<\frac{\left(n+1\right)\left(2+1/n\right)}{\left(n+2\right)\left(1+1/n\right)}=\frac{2n+1}{n+2}<2. \end{array}$$

Obviously, *a*_2_ − *a*_1_ = 0, *a*_3_ − *a*_2_ > 0; thus we can deduce that *a*_*n*+1_ > *a*_*n*_(*n* > 1) and then {*a*_*n*_} is a monotone bounded sequence. Therefore, lim⁡_*n*→*∞*_ *a*_*n*_ exists. From (64) we obtain

(67) $$\begin{array}{c} \underset{n\to \infty }{\lim}a_{n}=\sqrt{2} \end{array}$$

and then

(68) 0.5an−1,n+an,nan+1,n+1=0.5an−1,n+an,nan−1,n+an,n=0.5an+1an+1⟶22, n⟶∞.

From the proof of Theorem 16, we can see $a_{n}<\sqrt{2}$. From this and (54), it is easy to show $\sin x_{n-1,n}<\sin x_{n,n}=\sqrt{2}/2$ for (48) or (49).

Property 4 implies that *T*_*n*_(1, *x*) = 1. In order to show the convergence of trigonometric polynomials *T*_*n*_(*f*, *x*), we need to discuss *T*_*n*_(sin, *x*) and *T*_*n*_(cos⁡, *x*).

By *w*(*x*)^2^ = 2*c*(*x*)*s*(*x*), we have

(69) sinx =(c(x)+w(x))∑i=02n−2Ti,n−1(x) =∑i=0n(0.5ai−2,n−1+ai−1,n−1)sn−i(x)wi(x)  +∑i=n+12n(ai−2,n−1+ai−1,n−1)w2n−i(x)ci−n(x),cos⁡x =(s(x)+w(x))∑i=02n−2Ti,n(u) =∑i=0n−1(ai−1,n−1+ai,n−1)sn−i(x)wi(x)  +∑i=n2n(ai−1,n−1+0.5ai,n−1)w2n−i(x)ci−n(x).

The node expression (48) is set in the light of (69).

**Theorem 17 .**

For the node expression (48), *T*_*n*_(sin, *x*) and *T*_*n*_(cos⁡, *x*) converge uniformly to sin*x* and cos⁡*x*, respectively, for *x* ∈ [0, *π*/2].

**Proof**

By (54) we have $a_{i-2,n-1}/a_{i-1,n-1}<a_{i-1,n-1}/a_{i,n-1}<\sqrt{2}$ and then 4*a*_*i*,*n*−1_^2^ > *a*_*i*−2,*n*−1_^2^. From this we have

(70) ai−1,n−1+ai,n−1+2ai,n−1ai,n−ai,n−12≥ai,n,i=1,2,…,n−1.

Therefore,

(71) ai−1,n−1+ai,n−1ai,n−cos⁡xi,n =1ai,nai−1,n−12−ai−2,n−1ai,nai−1,n−1+ai,n−1+2ai,n−1ai,n−ai,n−12 <ai−1,n−12−ai−2,n−1ai,nai,n2,i=1,2,…,n−1,cos⁡xi,n=sinx2n−i,n=0.5a2n−i−2,n−1+a2n−i−1,n−1a2n−i,n=0.5ai,n−1+ai−1,n−1ai,n,i=n+1,n+2,…,2n

and then

(72) cos⁡x−Tn(cos⁡,x) =∑i=0n−1(ai−1,n−1+ai,n−1ai,n−cos⁡xi,n)Ti,n(x)  +(an−1,n−1+0.5an,n−1an,n−22)Tn,n(x) <∑i=0n−1ai−1,n−12−ai−2,n−1ai,n−1ai,n2Ti,n(x)  +(an−1,n−1+0.5an,n−1an,n−22)Tn,n(x).

In the same way, we have

(73) sinx−Tn(sin,x) =(0.5an−2,n−1+an−1,n−1an,n−22)Tn,n(x)  +∑i=n+12n(ai−2,n−1+ai−1,n−1ai,n−sinxi,n)Ti,n(x) <(0.5an−2,n−1+an−1,n−1an,n−22)Tn,n(x)  +∑i=n+12nai−1,n−12−ai−2,n−1ai,n−1ai,n2Ti,n(x).

From (57) and (58), it is easy to see that *T*_*n*_(sin, *x*) and *T*_*n*_(cos⁡, *x*) converge uniformly to sin*x* and cos⁡*x*, respectively, for *x* ∈ [0, *π*/2].

From the monotonicity of {*a*_*n*_}, we can know that

(74) $$\begin{array}{c} \frac{0.5a_{n-2,n-1}+a_{n-1,n-1}}{a_{n,n}}=\frac{a_{n-1,n-1}+0.5a_{n,n-1}}{a_{n,n}}>\frac{\sqrt{2}}{2}. \end{array}$$

Hence, with nodes (48), sin*x* > *T*_*n*_(sin, *x*) and cos⁡*x* > *T*_*n*_(cos⁡, *x*) for *x* ∈ (0, *π*/2).

Based on (69), if we minimize

(75) (sinxi,n−0.5ai−2,n−1+ai−1,n−1ai,n)2 +(cos⁡xi,n−ai−1,n−1+ai,n−1ai,n)2

for *i* = 0,1,…, *n* − 1, then the results are (53). If we minimize

(76) (sinxi,n−ai−2,n−1+ai−1,n−1ai,n)2 +(cos⁡xi,n−ai−1,n−1+0.5ai,n−1ai,n)2

for *i* = *n* + 1, *n* + 2,…, 2*n*, then

(77) $$\begin{array}{c} \tan x_{i,n}=\frac{a_{i-2,n-1}+a_{i-1,n-1}}{a_{i-1,n-1}+0.5a_{i,n-1}}. \end{array}$$

For (53) and (77), it is easy to validate that

(78) $$\begin{array}{c} x_{i,n}=\frac{\pi }{2}-x_{2n-i,n},\;i=n+1,n+2,\ldots ,2n. \end{array}$$

The node expression (49) is set in the light of the results of the minimality.

**Theorem 18 .**

For nodes (49), *T*_*n*_(sin, *x*) and *T*_*n*_(cos⁡, *x*) converge uniformly to sin*x* and cos⁡*x*, respectively, for *x* ∈ [0, *π*/2].

**Proof**

From (69), we have

(79) sinx−Tn(sin,x) =∑i≠nδi,nsinxi,nTi,n(x)  +(0.5an−2,n−1+an−1,n−1an,n−22)Tn,n(x),cos⁡x−Tn(cos⁡,x) =∑i≠nδi,ncos⁡xi,nTi,n(x)  +(0.5an−2,n−1+an−1,n−1an,n−22)Tn,n(x),

where

(80) δi,n=ai−1,n−12−ai−2,n−1ai,n−1ai,n(ai,n+Ai,n),Ai,n=(ai−2,n−1+ai−1,n−1)2+(ai−1,n−1+0.5ai,n−1)2,  i=n+1,n+2,…,2n.

Obviously,

(81) $$\begin{array}{c} 0\leq \delta_{i,n}<\frac{a_{i-1,n-1}^{2}-a_{i-2,n-1}a_{i,n-1}}{a_{i,n}^{2}}. \end{array}$$

Therefore, by (57) and (58), *T*_*n*_(sin*x*, *x*) and *T*_*n*_(cos⁡*x*, *x*) converge uniformly to sin*x* and cos⁡*x*, respectively, for *x* ∈ [0, *π*/2].

Obviously, with nodes (49), sin*x* > *T*_*n*_(sin, *x*) and cos⁡*x* > *T*_*n*_(cos⁡, *x*) for *x* ∈ (0, *π*/2).

In the following, for the sake of simplicity, we set *x*_*i*_ = *x*_*i*,*n*_ if it does not make a confusion.

**Theorem 19 .**

With nodes (48) or (49), the sequence of trigonometric polynomials *T*_*n*_(*f*, *x*) converges uniformly to *f* for all *f* ∈ *C*[0, *π*/2].

**Proof**

The proof is similar to the one used in proving Korovkin theorem; see [3, 20]. Let *ε* > 0; we want to prove that an integer *m* exists such that

(82) $$\begin{array}{c} n\geq m⟹||f-T_{n}f||<2\varepsilon , \end{array}$$

where

(83) $$\begin{array}{c} ||f-T_{n}f||=\underset{0\leq x\leq \pi /2}{\max}\left|f\left(x\right)-T_{n}\left(f,x\right)\right|. \end{array}$$

Since *f* is continuous on a compact interval, it is uniformly continuous. Consequently, a positive *δ* exists such that for all *x*_*i*_ and *x* in [0, *π*/2],

(84) $$\begin{array}{c} \left|x-x_{i}\right|<\delta ⟹\left|f\left(x\right)-f\left(x_{i}\right)\right|<\varepsilon . \end{array}$$

Let *φ*(*t*) = sin^2^(*t* − *x*)/2; we have

(85) $$\begin{array}{c} \left|x-x_{i}\right|\geq \delta ⟹\left|f\left(x\right)-f\left(x_{i}\right)\right|\leq 2||f||\leq \frac{2||f||}{\sin^{2}\delta /2}\varphi \left(x_{i}\right). \end{array}$$

Thus, for all *x*_*i*_ and *x* in [0, *π*/2], we have

(86) $$\begin{array}{c} \left|f\left(x\right)-f\left(x_{i}\right)\right|\leq \varepsilon +\frac{2||f||}{\sin^{2}\delta /2}\varphi \left(x_{i}\right) \end{array}$$

and then

(87) $$\begin{array}{c} \left|f\left(x\right)-T_{n}\left(f,x\right)\right|\leq \varepsilon +\frac{2||f||}{\sin^{2}\delta /2}T_{n}\left(\varphi ,x\right). \end{array}$$

Since *φ*(*t*) = (1 − cos⁡*x*cos⁡*t* − sin*x*sin*t*)/2, then

(88) $$\begin{array}{c} T_{n}\left(\varphi ,x\right)=\frac{1-\cos xT_{n}\left(\cos,x\right)-\sin xT_{n}\left(\sin,x\right)}{2}; \end{array}$$

thus the sequence *T*_*n*_(*φ*, *x*) converges uniformly to 0. Therefore, we can select *m* so that

(89) $$\begin{array}{c} \frac{2||f||}{\sin^{2}\delta /2}T_{n}\left(\varphi ,x\right)\leq \varepsilon \end{array}$$

whenever *n* ≥ *m*. Then ||*f* − *T*_*n*_ *f*||<2*ε*.

**Theorem 20 .**

Let *R*_*n*_(*f*, *x*) = *f*(*x*) − *T*_*n*_(*f*, *x*). If *f* ∈ *C* ^2^[0, *π*/2], then

(90) $$\begin{array}{c} \left|R_{n}\left(f,x\right)\right|\leq \sqrt{2}\left(2||f||+||f^{\prime }||+||f^{\prime \prime }||\right)R_{n}^{*}, \end{array}$$

where

(91) $$\begin{array}{c} R_{n}^{*}=\max\left\{||R_{n}\left(\sin,x\right)||,||R_{n}\left(\cos,x\right)||\right\}. \end{array}$$

**Proof**

It is easy to validate that

(92) f(xi)=f(x)cos⁡(xi−x)+f′(x)sin(xi−x) +∫xxisin(xi−t)[f′′(t)+f(t)]dt.

Let *x* ∈ [*x*_*k*_, *x*_*k*+1_]; then

(93) Tn(f,x)=[f(x)cos⁡x−f′(x)sinx]Tn(cos⁡,x) +[f(x)sinx+f′(x)cos⁡x]Tn(sin,x) +∑i=02nTi,n(x)∫xxisin(xi−t)[f′′(t)+f(t)]dt=f(x)−[f(x)cos⁡x−f′(x)sinx]Rn(cos⁡,x) −[f(x)sinx+f′(x)cos⁡x]Rn(sin,x) +∑i=0kTi,n(x)∫x0xsin(t−xi)+[f′′(t)+f(t)]dt +∑i=k+12nTi,n(x)∫xx2nsin(xi−t)+[f′′(t)+f(t)]dt,

where (*t* − *x*_*i*_)_+_ is the truncated power function. Since ∑_*i*=0_^*k*^ *T*_*i*,*n*_(*x*)sin(*t* − *x*_*i*_)_+_ is of one sign for *t* ∈ [*x*_0_, *x*], and ∑_*i*=*k*+1_^2*n*^ *T*_*i*,*n*_(*x*)sin(*x*_*i*_ − *t*)_+_ is of one sign for *t* ∈ [*x*, *x*_2*n*_], we have

(94) Rn(f,x)=[f(x)cos⁡x−f′(x)sinx]Rn(cos⁡,x) +[f(x)sinx+f′(x)cos⁡x]Rn(sin,x) −[f′′(η)+f(η)]∑i=0kTi,n(x)∫x0xsin(t−xi)+dt −[f′′(ξ)+f(ξ)]∑i=k+12nTi,n(x)∫xx2nsin(xi−t)+dt

for some *η* ∈ [*x*_0_, *x*] and *ξ* ∈ [*x*, *x*_2*n*_]. Therefore

(95) |Rn(f,x)|≤2(||f||+||f′||)Rn∗+(||f||+||f′′||) ×∑i=02nTi,n(x)[1−cos⁡(x−xi)]=2(||f||+||f′||)Rn∗+(||f||+||f′′||) ×[cos⁡xRn(cos⁡,x)+sinxRn(sin,x)]≤2(2||f||+||f′||+||f′′||)Rn∗.

### 3.3. The Convergence of the Derivative Functions

For the trigonometric polynomial (47), obviously,

(96) $$\begin{array}{c} T_{n}^{\prime }\left(f,x\right)=\overset{2n}{\underset{i=0}{\sum }}T_{i,n}^{\prime }\left(x\right)f\left(x_{i}\right). \end{array}$$

By Property 8, we obtain

(97) $$\begin{array}{c} T_{n}^{\prime }\left(f,x\right)=\overset{2n}{\underset{i=0}{\sum }}T_{i,n}\left(x\right)f^{\left[1\right]}\left(x_{i}\right), \end{array}$$

where

(98) f[1](xi)=(i+1)ai+1,nai,nf(xi+1)−(n−i)f(xi) −(2n−i+1)ai−1,n2ai,nf(xi−1)

for *i* = 0,1,…, *n* − 1,

(99) f[1](xn)=(n+1)an−1,n2an,n[f(xn+1)−f(xn−1)],f[1](xi)=(i+1)ai+1,n2ai,nf(xi+1)+(i−n)f(xi) −(2n−i+1)ai−1,nai,nf(xi−1)

for *i* = *n* + 1, *n* + 2,…, 2*n*. By Property 11, we have

(100) f[1](xi)=(i+1)ai+1,nai,n[f(xi+1)−f(xi)] +(2n−i+1)ai−1,n2ai,n[f(xi)−f(xi−1)]

for *i* = 0,1,…, *n* − 1, and

(101) f[1](xi)=(i+1)ai+1,n2ai,n[f(xi+1)−f(xi)] +(2n−i+1)ai−1,nai,n[f(xi)−f(xi−1)]

for *i* = *n* + 1, *n* + 2,…, 2*n*.

**Theorem 21 .**

With node expression (49), if *f* ∈ *C* ^2^[0, *π*/2], then the sequence of trigonometric polynomials *T*_*n*_′(*f*, *x*) converges uniformly to *f*′(*x*) for all *x* ∈ [0, *π*/2].

**Proof**

Let

(102) f[1](xi)=bi,nf′(xi)+ci,n=f′(xi)+(bi,n−1)f′(xi)+ci,n

for *i* = 0,1,…, 2*n*, we have

(103) Tn′(f,x)=∑i=02nTi,n(x)f′(xi) +∑i=02nTi,n(x)[(bi,n−1)f′(xi)+ci,n],|∑i=02nTi,n(x)[(bi,n−1)f′(xi)+ci,n]| ≤max⁡0≤i≤2n{|(bi,n−1)f′(xi)+ci,n|}.

Therefore, based on Theorem 19 and symmetry, we need only to show that *b*_*i*,*n*_ → 1 and *c*_*i*,*n*_ → 0 when *n* → *∞* for *i* = 0,1,…, *n*.

It is easy to show

(104) f(xi+1)=f(xi)cos⁡(xi+1−xi)+f′(xi)sin(xi+1−xi) +∫xixi+1sin(xi+1−t)[f′′(t)+f(t)]dt

for *i* = 0,1,…, *n* − 1, and

(105) f(xi−1)=f(xi)cos⁡(xi−xi−1)−f′(xi)sin(xi−xi−1) +∫xi−1xisin(t−xi−1)[f′′(t)+f(t)]dt

for *i* = 1,2,…, *n*. Thus, we have

(106) $$\begin{array}{c} b_{0,n}=n\sin\left(x_{1}-x_{0}\right),\\ c_{0,n}=n\left[f^{\prime \prime }\left(\eta_{0}\right)+f\left(\eta_{0}\right)-f\left(x_{0}\right)\right]\left[1-\cos\left(x_{1}-x_{0}\right)\right], \end{array}$$

for some *η*_0_ ∈ [*x*_0_, *x*_1_],

(107) bi,n=(i+1)ai+1,nai,nsin(xi+1−xi) +(2n−i+1)ai−1,n2ai,nsin(xi−xi−1),ci,n=(i+1)ai+1,nai,n[f′′(ηi)+f(ηi)−f(xi)] ×[1−cos⁡⁡(xi+1−xi)] +(2n−i+1)ai−1,n2ai,n[f(xi)−f′′(ξi)−f(ξi)] ×[1−cos⁡⁡(xi−xi−1)],

for some *η*_*i*_ ∈ [*x*_*i*_, *x*_*i*+1_], *ξ*_*i*_ ∈ [*x*_*i*−1_, *x*_*i*_], *i* = 1,2,…, *n* − 1, and

(108) bn,n=(n+1)an−1,nan,nsin(xn−xn−1),cn,n=(n+1)an−1,n2an,n ×{[f′′(ηn)+f(ηn)]  ×[1−cos⁡⁡(xn+1−xn)]  −[f′′(ξn)+f(ξn)][1−cos⁡(xn−xn−1)]},

for some *η*_*n*_ ∈ [*x*_*n*_, *x*_*n*+1_], *ξ*_*n*_ ∈ [*x*_*n*−1_, *x*_*n*_].

Obviously, when *n* → *∞*,

(109) b0,n=nn2+1⟶1,c0,n=n(1−nn2+1)[f′′(η0)+f(η0)−f(x0)]=nn2+nn2+1+1[f′′(η0)+f(η0)−f(x0)]⟶0.

For *i* = 1,2,…, *n* − 2, let

(110) $$\begin{array}{c} b_{i,n}=\frac{a_{i,n}a_{i+1,n}}{A_{i,n}A_{i+1,n}}d_{i,n}+\frac{a_{i-1,n}a_{i,n}}{A_{i-1,n}A_{i,n}}e_{i,n}, \end{array}$$

where

(111) $$\begin{array}{c} 0<d_{i,n}=\frac{i+1}{a_{i,n}^{2}}A_{i,n}A_{i+1,n}\sin\left(x_{i+1}-x_{i}\right),\\ 0<e_{i,n}=\frac{2n-i+1}{2a_{i,n}^{2}}A_{i-1,n}A_{i,n}\sin\left(x_{i}-x_{i-1}\right). \end{array}$$

Since

(112) ai−1,n−1ai,n−1−ai−2,n−1ai+1,n−1=ai−1,n−1i[(n−i)ai−1,n−1+0.5(2n−i)ai−2,n−1] −ai−2,n−1i+1[(n−i−1)ai,n−1+0.5(2n−i−1)ai−1,n−1]=n−ii(ai−1,n−12−ai−2,n−1ai,n−1) +ni(i+1)ai−2,n−1(ai−1,n−1+ai,n−1),

using (59), we have

(113) Ai,nAi+1,nsin(xi+1−xi) =(0.5ai−1,n−1+ai,n−1)(ai−1,n−1+ai,n−1)  −(0.5ai−2,n−1+ai−1,n−1)(ai,n−1+ai+1,n−1) =0.5(ai−1,n−12−ai−2,n−1ai,n−1)+ai,n−12−ai−1,n−1ai+1,n−1  +0.5(ai−1,n−1ai,n−1−ai−2,n−1ai+1,n−1) =ni(i+1)(0.5ai−2,n−1+ai−1,n−1)(ai−1,n−1+ai,n−1)  −n−i2i(ai−1,n−12−ai−2,n−1ai,n−1), =n2i(i+1)(ai−2,n−1+ai−1,n−1)(ai−1,n−1+2ai,n−1)  −n−i−12(i+1)(ai−1,n−12−ai−2,n−1ai,n−1)

and then, using (59),

(114) Ai−1,nAi,nsin(xi−xi−1) =ni(i−1)(0.5ai−3,n−1+ai−2,n−1)(ai−2,n−1+ai−1,n−1)  −n−i+12(i−1)(ai−2,n−12−ai−3,n−1ai−1,n−1) =ni(i−1)(12ai−3,n−1+n2n−i+1ai−2,n−1)  ×(ai−2,n−1+ai−1,n−1)  +n−i+12n−i+1(ai−1,n−12−ai−2,n−1ai,n−1) =ni(2n−i+1)(ai−2,n−1+ai−1,n−1)2  +n−i+12n−i+1(ai−1,n−12−ai−2,n−1ai,n−1).

From these we obtain

(115) $$\begin{array}{c} d_{i,n}+e_{i,n}=1+\frac{a_{i-1,n-1}^{2}-a_{i-2,n-1}a_{i,n-1}}{a_{i,n}^{2}}⟶1,\;n⟶\infty . \end{array}$$

Since *d*_*i*,*n*_ and *e*_*i*,*n*_ are bounded and

(116) Ai,nai,n=1+ai−1,n−12−ai−2,n−1ai,n−1ai,n2⟶1, n⟶∞,bi,n=(ai,nai+1,nAi,nAi+1,n−1)di,n+(ai−1,nai,nAi−1,nAi,n−1)ei,n +di,n+ei,n

we can conclude that *b*_*i*,*n*_ → 1 when *n* → *∞*.

By (44), we have

(117) sin(xi+1−xi)<n(0.5ai−2,n−1+ai−1,n−1)(ai−1,n−1+ai,n−1)i(i+1)Ai,nAi+1,n=ai,nai+1,n(0.5ai−2,n−1+ai−1,n−1)nAi,nAi+1,n(ai−2,n−1+ai−1,n−1)<1n

and then

(118) |ci,n|≤(2||f||+||f′′||) ×[(i+1)ai+1,nai,nsin2(xi+1−xi)   +(2n−i+1)ai−1,n2ai,nsin2(xi−xi−1)]≤1n2ai,n(2||f||+||f′′||) ×[(i+1)ai+1,n+0.5(2n−i+1)ai−1,n]=1n2ai,n(2||f||+||f′′||) ×[(n−i)ai,n+(2n−i+1)ai−1,n]<n−i+2(2n−i+1)n2(2||f||+||f′′||).

This implies that *c*_*i*,*n*_ → 0 when *n* → *∞*.

Using (44) repeatedly, we have

(119) sin(xn−1−xn−2)=1(n+2)An−2,nAn−1,n ×[nn−1(an−3,n−1+an−2,n−1)2   +2(an−2,n−12−an−3,n−1an−1,n−1)]=1(n+2)An−2,nAn−1,n ×[n−1nan−1,n2+2(an−2,n−12−an−3,n−1an−1,n−1)],sin(xn−xn−1)=22An−1,n(an−1,n−1−0.5an−3,n−1)=22An−1,n(an−1,n−an−3,n−1−an−2,n−1)=2an−1,n2nAn−1,n.

Thus we have

(120) bn−1,n=nan,nan−1,nsin(xn−xn−1) +(n+2)an−2,n2an−1,nsin(xn−1−xn−2)=2an,n2an−1,nan−1,nAn−1,n+(n−1)an−2,nan−1,n2nAn−2,nAn−1,n +an−2,n(an−2,n−12−an−3,n−1an−1,n−1)An−2,nan−1,nAn−1,n,bn,n=(n+1)an−1,nan,nsin(xn−xn−1)=2an−1,n2an,n(n+1)an−1,nnAn−1,n,|cn−1,n|≤(2||f||+||f′′||) ×[nan,nan−1,nsin2(xn−xn−1)   +(n+2)an−2,n2an−1,nsin2(xn−1−xn−2)]<1n2an−1,n(2||f||+||f′′||)[nan,n+0.5(n+2)an−2,n]=1n2[n−1+(n+2)an−2,nan−1,n](2||f||+||f′′||),|cn,n|≤(n+1)an−1,nan,n(||f||+||f′′||)sin2(xn−xn−1)=(n+1)an−1,n32n2an,nAn−1,n2(||f||+||f′′||).

Therefore,

(121) $$\begin{array}{c} \underset{n\to \infty }{\lim}b_{n-1,n}=1,\;\;\underset{n\to \infty }{\lim}b_{n,n}=1,\\ \underset{n\to \infty }{\lim}c_{n-1,n}=0,\;\;\underset{n\to \infty }{\lim}c_{n,n}=0. \end{array}$$

## 4. Quasi-Interpolation by the Trigonometric Polynomials

Like Bernstein polynomials, the convergence of the given trigonometric polynomials is slow. For reproducing one degree of trigonometric polynomials, we consider the following quasi-interpolant:

(122) Q(f,x)=∑i=02nTi,n(x)[αif(xi−1)+(1−αi−βi)×f(xi)+βif(xi+1)],

where *α*_0_ = *α*_2*n*_ = *β*_0_ = *β*_2*n*_ = 0.

Based on (69), in order to reproduce one degree of trigonometric polynomials by (122), we need to choose *α*_*i*_ and *β*_*i*_ to satisfy the following equalities:

(123) αisinxi−1+(1−αi−βi)sinxi+βisinxi+1  =1ai,n(0.5ai−2,n−1+ai−1,n−1),αicos⁡xi−1+(1−αi−βi)cos⁡xi+βicos⁡xi+1 =1ai,n(ai−1,n−1+ai,n−1),

for *i* = 1,2,…, *n* − 1,

(124) αnsinxn−1+(1−αn−βn)sinxn+βnsinxn+1 =1an,n(0.5an−2,n−1+an−1,n−1),αncos⁡xn−1+(1−αn−βn)cos⁡xn+βncos⁡xn+1 =1an,n(an−1,n−1+0.5an,n−1),αisinxi−1+(1−αi−βi)sinxi+βisinxi+1 =1ai,n(ai−2,n−1+ai−1,n−1),αicos⁡xi−1+(1−αi−βi)cos⁡xi+βicos⁡xi+1 =1ai,n(ai−1,n−1+0.5ai,n−1),

for *i* = *n* + 1, *n* + 2,…, 2*n* − 1.

Solving the above equations, for *i* = 1,2,…, *n* − 1, we have

(125) 2αisinxi−xi−12sinxi+1−xi−12−cos⁡xi+1−xi2 =−1ai,n[(0.5ai−2,n−1+ai−1,n−1)sinxi+xi+12     +(ai−1,n−1+ai,n−1)cos⁡xi+xi+12],2βisinxi+1−xi2sinxi+1−xi−12−cos⁡xi−xi−12 =−1ai,n[(0.5ai−2,n−1+ai−1,n−1)sinxi−1+xi2     +(ai−1,n−1+ai,n−1)cos⁡xi−1+xi2].

Using the node expression (49), we obtain $\alpha_{1}=\beta_{1}=-\sqrt{2}/2$ for *n* = 1, and

(126) $$\begin{array}{c} \alpha_{i}=-\delta_{i,n}\frac{\cos\left((x_{i+1}-x_{i})/2\right)}{2\sin\left(\left(x_{i}-x_{i-1}\right)/2\right)\sin\left(\left(x_{i+1}-x_{i-1}\right)/2\right)},\\ \beta_{i}=-\delta_{i,n}\frac{\cos(\left(x_{i}-x_{i-1}\right)/2)}{2\sin\left(\left(x_{i+1}-x_{i}\right)/2\right)\sin\left(\left(x_{i+1}-x_{i-1}\right)/2\right)}, \end{array}$$

for *n* > 1, *i* = 1,2,…, *n* − 1.

In the same way, let

(127) $$\begin{array}{c} A_{n,n}=\sqrt{2}\left(0.5a_{n-2,n-1}+a_{n-1,n-1}\right),\\ \delta_{n,n}=\frac{a_{n-1,n-1}^{2}-0.5a_{n-2,n-1}^{2}}{a_{n,n}\left(a_{n,n}+A_{n,n}\right)}; \end{array}$$

then (126) also holds for *n* > 1, *i* = *n*, *n* + 1,…, 2*n*. Thus, with all the coefficients *α*_*i*_ and *β*_*i*_, the quasi-interpolant (122) reproduces one degree of trigonometric polynomials.

**Example 22 .**

Let us consider the quasi-interpolant (122) for the functions *f*_1_ and *f*_2_. For *n* = 2, we have

(128) $$\begin{array}{c} \alpha_{1}=\beta_{3}=-\frac{1}{4}\left(\sqrt{2}+\sqrt{5}-1\right),\\ \alpha_{2}=\beta_{2}=-\frac{1}{4}\left(15\sqrt{2}+9\sqrt{5}-6\sqrt{10}-20\right),\\ \alpha_{3}=\beta_{1}=\sqrt{2}\alpha_{1}. \end{array}$$

**Figure 4:**
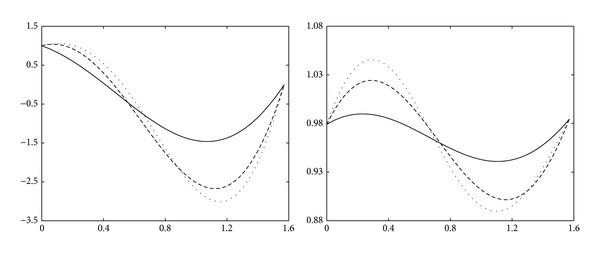
Quasi-interpolation for the functions *f*_1_ and *f*_2_.

On the left of Figure 4, for the function *f*_1_, the functional curve (dotted line), the quadratic trigonometric curve with node expression (49) (solid line), and the quasi-interpolation curve of quadratic trigonometric polynomial (dashed line) are shown, respectively. On the right of Figure 4, for the function *f*_2_, the functional curve (dotted line), the quadratic trigonometric curve with node expression (49) (solid line), and the quasi-interpolation curve of quadratic trigonometric polynomial (dashed line) are shown, respectively. Obviously, the quasi-interpolation of trigonometric polynomial approximates the functions better than the trigonometric polynomial does.

**Theorem 23 .**

With node expression (49) and the coefficients (126) for *i* = 1,2,…, 2*n* − 1, if *f* ∈ *C* ^2^[0, *π*/2], then the sequence of the quasi-interpolation trigonometric polynomials *Q*_*n*_(*f*, *x*) converges uniformly to *f*(*x*) for all *x* ∈ [0, *π*/2].

**Proof**

Obviously,

(129) $$\begin{array}{c} \alpha_{i}\sin\left(x_{i}-x_{i-1}\right)=\beta_{i}\sin\left(x_{i+1}-x_{i}\right),\\ \alpha_{i}\left[1-\cos\left(x_{i}-x_{i-1}\right)\right]+\beta_{i}\left[1-\cos\left(x_{i+1}-x_{i}\right)\right]=-\delta_{i,n}. \end{array}$$

By (104) and (105), we have

(130) αif(xi−1)+(1−αi−βi)f(xi)+βif(xi+1) =f(xi)+δi,n[f(xi)−f(ξi)−f′′(ξi)]

for some *ξ*_*i*_ ∈ [*x*_*i*−1_, *x*_*i*+1_]. From this we get

(131) Qn(f,x)=Tn(f,x) +∑i=12n−1Ti,n(x)δi,n[f(xi)−f(ξi)−f′′(ξi)].

Since

(132) $$\begin{array}{c} \underset{n\to \infty }{\lim}\delta_{i,n}=0, \end{array}$$

we have

(133) $$\begin{array}{c} \underset{n\to \infty }{\lim}Q_{n}\left(f,x\right)=\underset{n\to \infty }{\lim}T_{n}\left(f,x\right)=f\left(x\right). \end{array}$$

Based on the reproducing property of *Q*_*n*_(*f*, *x*), we can give an error expression. By

(134) f(x)=f(0)cos⁡x+f′(0)sinx +∫0xsin(x−t)[f′′(t)+f(t)]dt,

we have

(135) f(x)−Qn(f,x) =∫0xsin(x−t)[f′′(t)+f(t)]dt  −∑i=02nTi,n(x)     ×{αi∫0xi−1sin⁡(xi−1−t)[f′′(t)+f(t)]dt       +(1−αi−βi)       ×∫0xisin⁡(xi−t)[f′′(t)+f(t)]dt       +βi∫0xi+1sin(xi+1−t)[f′′(t)+f(t)]dt} =∫0π/2K(x,t)[f′′(t)+f(t)]dt,

where

(136) K(x,t)=sin⁡(x−t)+−∑i=02nTi,n(x)×[αisin(xi−1−t)++(1−αi−βi)sin(xi−t)++βisin(xi+1−t)+].

## 5. Conclusion

A symmetric basis of trigonometric polynomial space and its some interesting properties are presented. Using the positive trigonometric basis, symmetric trigonometric polynomial approximants are constructed. The trigonometric polynomial is simple and evident and easy for numerical computing. We are also interested in the particular basis and the trigonometric polynomial approximants because a constructive proof of trigonometric polynomial sequence approximating continuous function is given. The trigonometric polynomials have similar properties to Bernstein polynomials. Two kinds of node sequences are chosen particularly to show the convergence. We show that if a function is continuous on the interval [0, *π*/2] then the sequence of the trigonometric polynomials converges uniformly to the function on [0, *π*/2]. The derivative sequence of the trigonometric polynomials is also convergent if the function is twice differentiable. The trigonometric quasi-interpolants of reproducing one degree of trigonometric polynomials are constructed and the sequence is uniform convergent.

## Figures and Tables

**Table 1:** The coefficients {*a*_*i*,*n*_} of the trigonometric polynomials.

*n* = 1						1	1	1					
*n* = 2					1	2	2	2	1				
*n* = 3				1	3	$\frac{9}{2}$	4	$\frac{9}{2}$	3	1			
*n* = 4			1	4	8	10	$\frac{17}{2}$	10	8	4	1		
*n* = 5		1	5	$\frac{25}{2}$	20	$\frac{45}{2}$	$\frac{37}{2}$	$\frac{45}{2}$	20	$\frac{25}{2}$	5	1	
*n* = 6	1	6	18	35	$\frac{195}{4}$	51	41	51	$\frac{195}{4}$	35	18	6	1
